# Enhancing atrial fibrillation risk prediction in an observational cohort of tobacco-exposed individuals: the role of pulmonary function tests, symptom scores, and imaging

**DOI:** 10.1186/s12931-025-03366-8

**Published:** 2025-10-21

**Authors:** Nicole Curtis, S. Mehdi Nouraie, Jiantao Pu, Joseph K Leader, Frank C Sciurba, Jessica Bon

**Affiliations:** 1 University of Pittsburgh School of Medicine, 3350 Terrace Street, Pittsburgh, PA 15261 USA; 2 University of Pittsburgh, 3350 Terrace Street, Pittsburgh, PA 15261 USA; 3 Wake Forest University School of Medicine, 475 Vine St, Winston-Salem, NC 27101 USA

**Keywords:** COPD, Atrial fibrillation, Risk prediction, DLco, Heart volume, Emphysema, MMRC

## Abstract

**Introduction:**

COPD is associated with an increased AFib-related morbidity and mortality. There are several AFib risk prediction models available, but none have been validated in the COPD population. Our study aims to identify spirometric and radiographic variables that are associated with an increased risk of AFib. Secondarily, we hope to determine if these associated variables improve the risk discrimination of established AFib risk prediction models in individuals with COPD.

**Methods:**

We evaluated 755 participants from a single center tobacco-exposed cohort at baseline. At this study visit, the following were performed: demographic, medical history, and symptom questionnaires, PFT, and CT imaging. We performed logistic regression analysis to determine cardiopulmonary variables associated with prevalent AFib. The multivariable analysis was adjusted for sex, age, number of pack years, BMI, self-reported heart failure, and anti-hypertensive medication use. Exposure variables that were statistically significant in the logistic regression analysis were added in succession to current AFib risk prediction models, CHA_2_DS_2_-VASc and CHARGE-AF, to create updated models. C-statistics were calculated for both risk scores alone as well as with each updated model.

**Results:**

DLco (OR 0.40, CI 0.18–0.86), heart volume (OR 13.12, CI 2.32–74.17), percentage of emphysema (OR 2.77, CI 1.04–7.40), and mMRC (OR 1.17, CI 1.02–1.35) were associated with prevalent AFib in the multivariable logistic regression analysis. When conducting the discrimination analysis of the AFib risk prediction scores, the addition of these cardiopulmonary variables improved CHARGE-AF, from C-statistic 0.53 to 0.63 (*p* < 0.03).

**Conclusions:**

We identified cardiopulmonary factors associated with an increased risk of AFib in a tobacco-exposed cohort. The incorporation of lung function, CT parameters, and symptom scores in validated AFib prediction models may improve AFib risk discrimination in our chronic lung disease populations.

**Clinical trial number:**

not applicable.

**Trial registration:**

This study was supported by the National Institute of Health (NIH) National Heart, Lung and Blood Institute (NHLBI) grants 1R01HL128289 (J.B.) and P50HL084948 (F.C.S.).

**Supplementary Information:**

The online version contains supplementary material available at 10.1186/s12931-025-03366-8.

## Introduction

Chronic obstructive pulmonary disease (COPD) is associated with extrapulmonary cardiovascular comorbidities including coronary heart disease, heart failure, and atrial fibrillation (AFib) [1, 2]. While the prevalence of AFib in the general adult population is approximately 3%, patients with COPD have a 28% increased risk of developing AFib [3]. Hypoxia, hypercapnia, pulmonary hypertension, electrolyte disturbances, oxidative stress, and chronic systemic inflammation are all proposed processes that contribute to this increased risk [4, 5]. In addition to the increased risk of developing AFib, patients with COPD have worse AFib-related morbidity and mortality [4–6]. Numerous studies have attempted to understand why COPD is associated with an increased risk of AFib, but there is a paucity of studies evaluating specific lung-related variables via spirometry and imaging.

There are numerous validated and commonly utilized risk prediction models to predict development of AFib, including CHA_2_DS_2_-VASc and the Cohorts for Heart and Aging Research in Genomic Epidemiology AF (CHARGE-AF). Both have not been validated in COPD populations [7–9]. A need exists to validate these prediction models in a COPD population, and to determine if factors related to lung disease severity can improve risk discrimination of these models in patients with COPD. Our study aims to identify spirometric and radiographic variables that are associated with an increased risk of AFib. Secondarily, we hope to determine if these associated variables improve the risk discrimination of established AFib risk prediction models in individuals with COPD. We hypothesize that spirometric and radiographic variables indicative of worse airflow obstruction, a greater percentage of emphysema, and larger heart volume are associated with increased risk of AFib and will improve risk discrimination of CHA_2_DS_2_-VASc and CHARGE-AF.

## Methods

### Study population

The COPD Specialized Center for Clinically Oriented Research (SCCOR) cohort at the University of Pittsburgh is a prospective observational cohort of 765 current or former smokers with at least a 10 pack-year history of tobacco use [10]. The cohort enrolled participants between July 2007 and December 2012. Exclusion criteria included history of lung cancer, obesity (defined as body mass index (BMI) >34), and any other significant pulmonary diagnosis. At each study visit, the following were performed: demographic, medical history, and symptom questionnaires, pulmonary function testing (PFT), and chest computed tomography (CT) imaging. For the analysis presented in this manuscript, we extracted the SCCOR data set in September 2023 and performed statistical analysis through November 2024. All 765 SCCOR participants were eligible to be included in our analysis. 10 participants were excluded due to missing data of the primary outcome (self-reported AFib) on medical history questionnaire. We analyzed baseline visit data only.

## Study procedures and definitions

Prevalent AFib was defined by self-report at the baseline visit. Modified Medical Research Council (mMRC) dyspnea scale was used to assess shortness of breath with scores ranging from 0 (no breathlessness) to 4 (severe breathlessness) [11]. PFTs were performed and included pre- and post- bronchodilator spirometry, lung volumes, and diffusion capacity of the lungs for carbon monoxide (DLco). Post-bronchodilator spirometry was performed using an albuterol metered dose inhaler with 2 inhalations 30 min before repeat testing. Body plethysmography (Model Vmax V62, SensorMedics/CareFusion Corp, Yorba Linda, CA) was performed. Lung diffusion capacity was measured by the single breath carbon monoxide (DLco) technique. Participants were required to have a minimum of three acceptable maneuvers with the two highest forced vital capacity (FVC) and forced expiratory volume in one second (FEV_1_) values within 150 mL to ensure reproducibility. Acceptable functional residual capacity (FRC) measurements had values within 5% of each other with the highest value reported if reproducibility criteria were met. The quality of each maneuver was graded A-F based on ATS/ERS (American Thoracic Society/European Respiratory Society) technical acceptability criteria. Expiratory reserve volume (ERV) was measured. Residual volume (RV) was calculated as FRC minus ERV. Total lung capacity (TLC) was calculated as RV plus vital capacity (VC). Global Lung Function Initiative (GLI) reference equations were used to calculate predicted values for spirometry adjusted by race [12], DLco [13], and static lung volumes [14].

A blinded radiologist performed quantitative scoring of CT scans. The lung parenchyma was automatically segmented from the chest wall and large central blood vessels using an in-house software. A density mask of −910 Hounsfield Units was applied as the emphysema threshold and the percentage of voxels below this threshold was calculated. The volume of cardiac and aortic calcifications was calculated using the Agatson score [15]. The diameter of major pulmonary vasculature and heart volume was computed automatically using an artificial intelligence (AI) algorithm. For heart volume, we used the U-Net mode, a widely used deep learning model, to segment heart volume from chest CT scans. Once the heart was segmented, the volume was computed by counting the number of voxels within the segmented region and multiplying by the voxel dimensions derived from the CT scan metadata, resulting in total heart volume expressed in liters [16]. For pulmonary vasculature, our radiologists used an in-house developed, previously published algorithm [17].

CHA_2_DS_2_-VASc and CHARGE-AF were calculated for each participant. CHA_2_DS_2_-VASc was calculated by totaling points for the following: heart failure (1 point), hypertension (1 point), age >75 (2 points), diabetes (1 point), stroke/transient ischemic attack/thromboembolism (2 points), vascular disease (1 point), age 65–74 (1 point), and female sex (1 point) [8]. Comorbidities were assessed with self-reported medical history questionnaires. CHARGE-AF was calculated utilizing an intricate formula cited in the 2023 ACC/AHA/ACCP/HRS guidelines including the following variables: age, race, height, weight, systolic blood pressure, diastolic blood pressure, smoking status, history of diabetes, and history of myocardial infarction [18].

### Statistical analysis

Prevalent AFib at baseline was the primary outcome of interest. Independent PFT variables included FEV_1_, FVC, FEV_1_/FVC, residual volume (RV), TLC, and DLco. Independent radiographic variables included quantitative emphysema, the ratio of pulmonary artery to aorta diameter, heart volume, and volume of vascular calcifications. mMRC was used to quantify symptom severity.

Comparison of baseline characteristics between participants with and without prevalent AFib were evaluated using the independent Student’s t-test for continuous variables and chi-square analysis for categorical variables. Time-varying confounding was not considered, as all data analyzed was collected at the baseline visit. Univariate and multivariable logistic regression was performed to determine cardiopulmonary variables associated with prevalent AFib. The multivariable analysis was adjusted for the following: sex, age, number of pack years, BMI, self-reported heart failure, and anti-hypertensive medication use. All cardiopulmonary variables were analyzed as continuous variables. DLco, RV/TLC ratio, and percentage of emphysema were additionally analyzed as binary variables with defined thresholds in the logistic regression.

Discrimination analysis was performed for both CHA_2_DS_2_-VASc and CHARGE-AF using the area under the time-dependent receiver operating characteristic curve (AUROC). Exposure variables that were statistically significant in the logistic regression analysis were added in succession to these risk prediction scores to create updated models. C-statistic was determined for CHA_2_DS_2_-VASc and CHARGE-AF alone as well as with each updated model.

All statistical analyses were performed via Stata version 18.0^19^. This study was conducted and reported in accordance with the Strengthening the Reporting of Observational Studies in Epidemiology (STROBE) guidelines (appendix 1)^20^.

## Results

We evaluated 755 participants from the SCCOR cohort, of whom 135 (17.9%) reported a history of AFib at the baseline visit (prevalent AFib).

Baseline age, sex, race, and pack years were similar among participants with and without prevalent AFib (Table 1). Those with prevalent AFib had a lower DLco, greater amount of emphysema, worse mMRC score, and larger heart volume. There was a higher percentage of self-reported diabetes, angina, heart failure, thromboembolic disease, hyperlipidemia, and anti-hypertensive medication use in the prevalent AFib group compared to no AFib.

**Table 1:** Baseline characteristics

	Prevalent AFib *N* = 135	No AFib *N* = 620	*p*-value
Demographics			
Age, years, mean (SD)	65.2 (7.0)	64.0 (7.0)	0.07
Male Sex, n (%)	63 (46.7%)	335 (54.0%)	0.12
Race, n (%) White Black Multi-Racial Unknown	113 (83.7%) 5 (3.7%) 1 (0.7%) 16 (11.9%)	525 (84.7%) 25 (18.5%) 3 (0.5%) 67 (10.8%)	0.92
Ethnicity, n (%) Non-Hispanic Hispanic Unknown	119 (88.1%) 0 16 (11.9%)	549 (88.5%) 4 (0.6%) 67 (10.8%)	0.35
Currently Smoke, n (%)	34 (25.2%)	215 (34.7%)	0.03
Pack Years, mean (SD)	58.1 (33.7)	54.1 (31.8)	0.20
Cardiopulmonary Variables			
FEV_1_% predicted, mean (SD)	0.67 (0.31)	0.69 (0.30)	0.51
FEV_1_/FVC < 70%, n (%)	83 (61.5%)	391 (63.1%)	0.77
DLco % predicted, mean (SD)	0.61 (0.25)	0.66 (0.26)	0.03
RV/TLC Ratio, mean (SD)	0.42 (0.09)	0.41 (0.09)	0.56
mMRC	1.67 (1.36)	1.41 (1.37)	0.04
% Emphysema	0.61 (0.49)	0.49 (0.50)	0.008
PA/PV Diameter Ratio	0.94 (0.16)	0.94 (0.16)	0.85
Heart Volume	0.54 (0.18)	0.51 (0.13)	0.02
Coronary Calcifications	0.64 (0.91)	0.65 (0.98)	0.91
Aorta Calcifications	4.74 (5.64)	3.95 (6.69)	0.22
Comorbidities, n (%)			
Myocardial Infarction	6 (4.4%)	24 (3.9%)	0.58
Diabetes	15 (11.1%)	31 (5%)	0.002
Angina	22 (16.3%)	53 (8.6%)	0.007
Heart Failure	4 (3.0%)	5 (0.8%)	0.04
Stroke	6 (4.4%)	27 (4.4%)	0.96
Thromboembolism	14 (10.4%)	26 (4.2%)	0.004
Anti-HTN Med	79 (58.5%)	228 (36.8%)	< 0.001
Hyperlipidemia	79 (58.5%)	302 (48.7%)	0.02

*SD* Standard deviation, *FEV 1* forced expiratory volume in one sec, *FVC* forced vital capacity, *DLco* diffusion capacity of the lungs for carbon monoxide, *mMRC* Modified Medical Research Council, *RV/TLC* residual volume/total lung capacity, *PA/PV* pulmonary artery/pulmonary vein diameter, *HTN* hypertension

### Association of Cardiopulmonary Variables with Prevalent AFib

 In the univariate analysis, DLco, heart volume, percentage of emphysema, and mMRC all showed statistically significant associations with prevalent AFib (Table 2). These associations remained significant when adjusting for sex, age, number of pack years, BMI, heart failure, and anti-hypertensive medication use. FEV₁ and the presence of airflow obstruction were not significantly associated with prevalent AFib.

**Table 2:** Association of cardiopulmonary variables with prevalent AFib

	Univariate OR (95% CI)	Multivariable OR (95% CI)
FEV_1_% predicted	0.81(0.44–1.51)	0.73 (0.38–1.40)
FEV_1_/FVC < 70%	0.94 (0.64–1.40)	0.92 (0.61–1.37)
DLco % predicted < 60% *≥* 60%	0.44 (0.21–0.93) 1.33 (0.91–1.94) Ref	0.40 (0.18–0.86) 1.37 (0.83-2.00) Ref
RV/TLC Ratio < 40% > 40%	1.57 (0.40–6.17) Ref 0.89 (0.60–1.31)	1.40 (0.33–5.92) Ref 0.79 (0.53–1.18)
mMRC	1.15 (1.004–1.31)	1.17 (1.02–1.35)
% Emphysema < 20% *≥* 20%	2.26 (0.87–5.83) Ref 1.45 (1.27–1.66)	2.77 (1.04–7.40) Ref 1.80 (1.22–2.66)
PA/PV Diameter Ratio	0.89 (0.26–2.99)	0.79 (0.22–2.83)
Heart Volume	4.67 (1.26–17.32)	13.12 (2.32–74.17)
Right Coronary Calcifications	2.30 (1.04–5.12)	2.19 (0.94–5.13)
Left Coronary Calcifications	0.93 (0.73–1.18)	0.889 (0.68–1.16)
Coronary Calcifications	0.99 (0.81–1.21)	0.96 (0.77–1.20)
Aorta Calcifications	1.02 (0.99–1.94)	1.005 (0.98–1.03)

*OR* odds ratio, *CI* confidence interval, *FEV*_*1*_ forced expiratory volume in one sec, *FVC* forced vital capacity, *DLco* diffusion capacity of the lungs for carbon monoxide, *RV/TLC* residual volume/total lung capacity, *mMRC* Modified Medical Research Council

### Cardiopulmonary Variables Improve Discrimination of AFib Risk Prediction Models

 There was a statistically significant difference in mean CHA_2_DS_2_-VASc between those with and without prevalent AFib. For CHARGE-AF, there was no difference between the two groups (Table 3).

**Table 3:** Mean AFib risk prediction scores

	Prevalent AFib *N* = 135	No AFib *N* = 620	*p*-value
CHA_2_DS_2_-VASc	2.28	1.68	< 0.001
CHARGE-AF	12.44	12.35	0.38

**Fig. 1:**
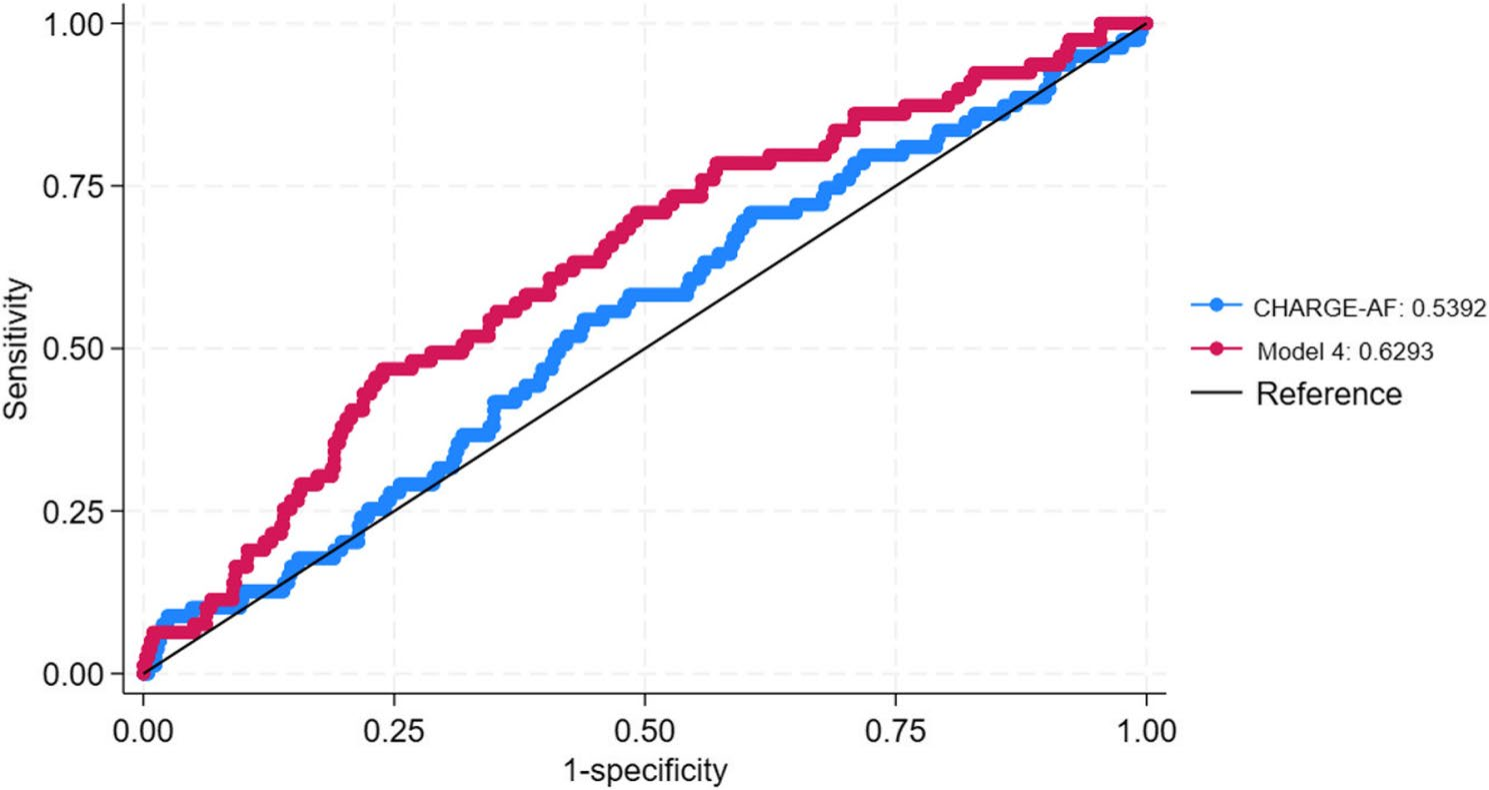
Discrimination of an updated model for preventing AFib risk prediction

When conducting the discrimination analysis for prevalent AFib, the C-statistic was greater for CHA2DS2-VASc than CHARGE-AF, indicating it as a better risk predictor in our study population. This improved with the sequential addition of our exposure variables. Model 4 had the greatest improvement of discrimination, most notably for CHARGE-AF; this model incorporated DLco, heart volume, degree of emphysema, and mMRC score (Table 4; Fig. 1).

**Table 4:** Discrimination of AFib risk prediction scores updated with cardiopulmonary variables

	Prevalent AFib C-Statistic (95% CI)	*p*-value*
CHA _2_ DS _2_ -VASc	0.62	
^a^Model 1	0.63 (0.57–0.68)	0.45
^b^Model 2	0.65 (0.60–0.70)	0.17
^c^Model 3	0.65 (0.60–0.71)	0.13
^d^Model 4	0.66 (0.60–0.71)	0.10
CHARGE-AF	0.53	
^a^Model 1	0.58 (0.51–0.65)	0.19
^b^Model 2	0.61 (0.54–0.67)	0.09
^c^Model 3	0.61 (0.54–0.68)	0.09
^d^Model 4	0.63 (0.56–0.70)	0.03

*CI *confidence interval

*p-value reflects comparison between each Model to the corresponding AF risk prediction model,* CHA*_*2*_*DS*_*2*_*-VASc or CHARGE-AF*

*Each model incorporates either CHA*_*2*_*DS*_*2*_*-VASc or CHARGE-AF plus the following*:

^*a*^*Model 1: DLco % predicted*

^*b*^*Model 2: DLco % predicted*,* heart volume*

^*c*^*Model 3: DLco % predicted*,* heart volume*,* % emphysema > 20%*,

^*d*^*Model 4: DLco % predicted*,* heart volume*,* % emphysema > 20%*,* mMRC score*

## Discussion

In our analysis, we found that lower DLco, larger heart volume, greater percentage of emphysema, and worse mMRC scores are associated with an increased risk of AFib in our tobacco-exposed cohort. We found that CHA_2_DS_2_-VASc was an overall better predictor of prevalent AFib than CHARGE-AF in our study population. The addition of cardiopulmonary physiologic and radiographic variables to these risk prediction models improves risk discrimination. Specifically, the incorporation of the Model 4 variables (DLco, heart volume, percentage of emphysema, and mMRC score) improved risk discrimination of CHARGE-AF to a greater degree compared to CHA_2_DS_2_-VASc. Both risk prediction models alone had overall worse discrimination than previously validated studies [7, 8], likely due to differences in study populations, with our study population having a significantly greater percentage of smokers and hypertension compared to those populations studied in CHA_2_DS_2_-VASc and CHARGE-AF. Moreover, while these two risk prediction models include similar variables in their calculations, they carry different weights. For CHA^2^DS^2^-Vasc, age >75 years and history of stroke or transient ischemic attack (TIA) carries a weight of 2 points compared to that of 1 for heart failure, hypertension, diabetes, vascular disease, age 65–74, and female sex. For CHARGE-AF, the highest coefficient in the algorithm is for heart failure and history of a myocardial infarction. This may account for the differing baseline discrimination for the two models.

Large cohort studies, including the Copenhagen City Heart Study, the Atherosclerosis Risk in Communities (ARIC) cohort study, and the Malmo Preventative project show that a lower FEV1 is associated with an increased risk of developing AFib [5]. While FEV1 was not shown to be statistically significant in our study population, we found that previously unstudied variables, including DLco, percentage of emphysema, heart volume, and mMRC are associated with prevalent AFib. Previous studies outline a pathway for these associations. Emphysema leads to DLco impairment and hypoxia which is known to contribute to AFib risk [4, 5]. Elevated right ventricle (RV) afterload, often from subsequent hypoxic vasoconstriction and remodeling, is also associated with an increased risk of AFib [21]. Atrial dilation likewise associates with AFib [5], and while not feasible to measure in our cohort, total heart volume was associated with risk of Afib in our cohort. Echocardiography and cardiac magnetic resonance imaging (MRI), though not available in our cohort, would have been useful measures to further highlight this relationship. Left ventricle (LV) and left atrial (LA) impairment [22] and the autonomic nervous system [23] have also been linked with AFib risk, further emphasizing the multifactorial etiologies of AFib.

The addition of cardiopulmonary variables to CHA2DS2-VASc and CHARGE-AF greatly improved risk discrimination, most pronounced in CHARGE-AF predicting prevalent AFib. While there are many AFib risk prediction models available, CHARGE-AF has been shown to have the best success in predicting AFib development in 5 years [24, 25]. Both CHA2DS2-VASc and CHARGE-AF scores are comprised of demographic data and cardiac comorbidities. Neither includes lung-related variables and, although both were derived from a diverse patient population of >10,000 individuals, they did not assess lung-related factors other than smoking status [7, 8]. Therefore, whether these scores are generalizable to a COPD population is unclear, and their relative low performance in our tobacco-exposed cohort would suggest that they are not. We have shown that the incorporation of additional cardiopulmonary variables to these validated risk scores improves AFib risk prediction in a COPD population.

Our analysis has several limitations to note. AFib was assessed via self-report rather than through electronic health record (EHR) review or EKG documentation. Since AFib is often asymptomatic and paroxysmal, our number of participants with AFib was likely an under-estimate of the true prevalence and can be a source of measurement bias. There are also likely unmeasured confounding variables that we can’t account for. We did not include incident AFib (new AFib diagnosed during our follow-up period) in our analysis due to concern for variation in results secondary to two mechanisms, (1) a high dropout rate likely reducing the reported incident AFib cases and (2) those who developed incident AFib at a later timepoint were healthier with a higher FEV1, less pack year history, and fewer obstructive PFTs.

Additionally, while we did not validate our findings in a separate cohort, this likely did not affect study results as the CHA2DS2-VASc and CHARGE-AF have already been validated in prior cohorts. Finally, our CT variables were created by an AI research algorithm and therefore may not be generalizable to a clinical population. Though we were able to evaluate chest CT scans, other radiographic studies like echocardiography or cardiac MRI were not available variables in the SCCOR cohort. Other variables, such as exacerbation history and active medication therapies could also be useful for analysis in future studies.

## Conclusion

We identified cardiopulmonary factors associated with an increased risk of AFib in a tobacco-exposed cohort. The incorporation of lung function, CT parameters, and symptom scores in validated AFib prediction models may improve AFib risk discrimination in our chronic lung disease populations.

## Supplementary Information

Supplementary Material 1.

## Data Availability

Data can be made available upon request by emailing the corresponding author.
