# Supplementary material for: Enhancing atrial fibrillation risk prediction in an observational cohort of tobacco-exposed individuals: the role of pulmonary function tests, symptom scores, and imaging
Source: Respir Res. 2025 Oct 21;26:287. doi: 10.1186/s12931-025-03366-8 (PMC12538955; doi:10.1186/s12931-025-03366-8)
Supplement: Supplementary file 1 — Supplementary Material 1. [file 12931_2025_3366_MOESM1_ESM.docx]

**Appendix 1. STROBE Statement**

|  | Item No. | Recommendation | Page  No. | Relevant text from manuscript |
| --- | --- | --- | --- | --- |
| **Title and abstract** | 1 | (*a*) Indicate the study’s design with a commonly used term in the title or the abstract | 1 | Enhancing Atrial Fibrillation Risk Prediction in an Observational Cohort of Tobacco-Exposed Individuals: The Role of Pulmonary Function Tests, Symptom Scores, and Imaging |
|  |  | (*b*) Provide in the abstract an informative and balanced summary of what was done and what was found | 2 | Included. |
| Introduction | | | |  |
| Background/rationale | 2 | Explain the scientific background and rationale for the investigation being reported | 3 | COPD is associated with an increased AFib-related morbidity and mortality. There are several AFib risk prediction models available, but none have been validated in the COPD population. Numerous studies have attempted to understand why COPD is associated with an increased risk of AFib, but there is a paucity of studies evaluating specific lung-related variables via spirometry and imaging. |
| Objectives | 3 | State specific objectives, including any prespecified hypotheses | 4 | Our study aims to identify spirometric and radiographic variables that are associated with an increased risk of AFib. Secondarily, we hope to determine if these associated variables improve the risk discrimination of established AFib risk prediction models in individuals with COPD. We hypothesize that spirometric and radiographic variables indicative of worse airflow obstruction, a greater percentage of emphysema, and larger heart volume are associated with increased risk of AFib and will improve risk discrimination of CHA_2_DS_2_-VASc and CHARGE-AF. |
| Methods | | | |  |
| Study design | 4 | Present key elements of study design early in the paper | 4 | The COPD Specialized Center for Clinically Oriented Research (SCCOR) cohort at the University of Pittsburgh is a prospective observational cohort of 765 current or former smokers with at least a 10 pack-year history of tobacco use. |
| Setting | 5 | Describe the setting, locations, and relevant dates, including periods of recruitment, exposure, follow-up, and data collection | 4 | Setting: Pittsburgh  Enrollment (for SCCOR): Study enrollment between July 2007 and December 2012.  Follow-up (for SCCOR): Baseline, 2, 6, 10-year follow up visits  Data analysis: For the analysis presented in this manuscript, we extracted the SCCOR data set in September 2023 and performed statistical analysis through November 2024. We analyzed baseline visit data only. |
| Participants | 6 | (*a*) *Cohort study*—Give the eligibility criteria, and the sources and methods of selection of participants. Describe methods of follow-up  *Case-control study*—Give the eligibility criteria, and the sources and methods of case ascertainment and control selection. Give the rationale for the choice of cases and controls  *Cross-sectional study*—Give the eligibility criteria, and the sources and methods of selection of participants | 4 | Eligibility criteria for SCCOR cohort included current or former smokers with at least a 10 pack-year history of tobacco use. Participants were evaluated at baseline (N=765), two (N=393), six (N=298), and ten year (N=101) follow up. For the analysis presented in this manuscript, we extracted the SCCOR data set in September 2023 and performed statistical analysis through November 2024. All 765 SCCOR participants were eligible to be included in our analysis. 10 participants were excluded due to missing data of the primary outcome (self-reported AFib) on medical history questionnaire. We analyzed baseline visit data only. |
|  |  | (*b*) *Cohort study*—For matched studies, give matching criteria and number of exposed and unexposed  *Case-control study*—For matched studies, give matching criteria and the number of controls per case | n/a | This was not a matched study. |
| Variables | 7 | Clearly define all outcomes, exposures, predictors, potential confounders, and effect modifiers. Give diagnostic criteria, if applicable | 5-6 | Prevalent AFib was defined by self-report at the baseline visit. Modified Medical Research Council (mMRC) dyspnea scale was used to assess shortness of breath with scores ranging from 0 (no breathlessness) to 4 (severe breathlessness)^11^. PFTs were performed at each study visit and included pre- and post- bronchodilator spirometry, lung volumes, and diffusion capacity of the lungs for carbon monoxide (DLco). Post-bronchodilator spirometry was performed using an albuterol metered dose inhaler with 2 inhalations 30 minutes before repeat testing. Body plethysmography (Model Vmax V62, SensorMedics/CareFusion Corp, Yorba Linda, CA) was performed. Lung diffusion capacity was measured by the single breath carbon monoxide (DLco) technique. Participants were required to have a minimum of three acceptable maneuvers with the two highest forced vital capacity (FVC) and forced expiratory volume in one second (FEV_1_) values within 150 mL to ensure reproducibility. Acceptable functional residual capacity (FRC) measurements had values within 5% of each other with the highest value reported if reproducibility criteria were met. The quality of each maneuver was graded A-F based on ATS/ERS (American Thoracic Society/European Respiratory Society) technical acceptability criteria. Expiratory reserve volume (ERV) was measured. Residual volume (RV) was calculated as FRC minus ERV. Total lung capacity (TLC) was calculated as RV plus vital capacity (VC). Global Lung Function Initiative (GLI) reference equations were used to calculate predicted values for spirometry adjusted by race^12^, DLco^13^, and static lung volumes^14^.  Chest CT scans were also obtained at each study visit. A blinded radiologist performed quantitative scoring of CT scans. The lung parenchyma was automatically segmented from the chest wall and large central blood vessels using an in-house software. A density mask of -910 Hounsfield Units was applied as the emphysema threshold and the percentage of voxels below this threshold was calculated. The volume of cardiac and aortic calcifications was calculated using the Agatson score^13^. The diameter of major pulmonary vasculature and heart volume was computed automatically using an artificial intelligence (AI) algorithm. For heart volume, we used the U-Net mode, a widely used deep learning model, to segment heart volume from chest CT scans. Once the heart was segmented, the volume was computed by counting the number of voxels within the segmented region and multiplying by the voxel dimensions derived from the CT scan metadata, resulting in total heart volume expressed in liters^14^. For pulmonary vasculature, our radiologists used an in-house developed, previously published algorithm^15^. |
| Data sources/ measurement | 8* | For each variable of interest, give sources of data and details of methods of assessment (measurement). Describe comparability of assessment methods if there is more than one group | 5-6 | Included above. |
| Bias | 9 | Describe any efforts to address potential sources of bias | 5 | We used uniform data collection methods, including standard demographic/medical questionnaire, well-described CT algorithm for radiographic variables, and standardized PFT procedures.  We had a low number of missing data. Only 10/765 (1.3%) had missing data for the primary outcome. |
| Study size | 10 | Explain how the study size was arrived at | 4 | We used a convenience sample. All members of the SCCOR cohort were eligible for inclusion. We excluded any participant with missing data for the primary outcome. 10 participants were excluded for this reason. |

Continued on next page

| Quantitative variables | 11 | Explain how quantitative variables were handled in the analyses. If applicable, describe which groupings were chosen and why | 6-7 | Comparison of baseline characteristics between participants with and without prevalent AFib were evaluated using the independent Student’s t-test for continuous variables and chi-square analysis for categorical variables. All cardiopulmonary variables were analyzed as continuous variables. DLco, RV/TLC ratio, and percentage of emphysema were additionally analyzed as binary variables with defined thresholds in the logistic regression. |
| --- | --- | --- | --- | --- |
| Statistical methods | 12 | (*a*) Describe all statistical methods, including those used to control for confounding | 6-7 | Comparison of baseline characteristics between participants with and without prevalent AFib were evaluated using the independent Student’s t-test for continuous variables and chi-square analysis for categorical variables. Time-varying confounding was not considered as all data was collected at the baseline visit. Univariate and multivariable logistic regression was performed to determine cardiopulmonary variables associated with prevalent AFib. The multivariable analysis was adjusted for the following: sex, age, number of pack years, BMI, self-reported heart failure, and anti-hypertensive medication use.  Discrimination analysis was performed for both CHA_2_DS_2_-VASc and CHARGE-AF using the area under the time-dependent receiver operating characteristic curve (AUROC). Exposure variables that were statistically significant in the logistic regression analysis were added in succession to these risk prediction scores to create updated models. C-statistic was determined for CHA_2_DS_2_-VASc and CHARGE-AF alone as well as with each updated model. |
|  |  | (*b*) Describe any methods used to examine subgroups and interactions | n/a | We did not utilize subgroup analysis. |
|  |  | (*c*) Explain how missing data were addressed | 4-5 | Missing data for the primary outcome (self-reported AFib) was assessed. Only 10/765 (1.3%) had missing data. These participants were excluded from the analysis. |
|  |  | (*d*) *Cohort study*—If applicable, explain how loss to follow-up was addressed  *Case-control study*—If applicable, explain how matching of cases and controls was addressed  *Cross-sectional study*—If applicable, describe analytical methods taking account of sampling strategy | 4-7 | All variables assessed in this study were retrieved from the baseline visit. |
|  |  | (*e*) Describe any sensitivity analyses | n/a | We did not conduct a sensitivity analysis. |
| Results | | | | |
| Participants | 13* | (a) Report numbers of individuals at each stage of study—eg numbers potentially eligible, examined for eligibility, confirmed eligible, included in the study, completing follow-up, and analysed | 4-5 | 765 participants were eligible for inclusion in our data analysis. 10 were excluded due to missing data from the primary outcome of self-reported AFib at baseline. In this data analysis we only analyzed baseline visit data. |
|  |  | (b) Give reasons for non-participation at each stage | 4-5 | Lost to follow for the SCCOR cohort was likely multifactorial including (1) prolonged study period (2) moving away from study center or (3) death or major illness. In this data analysis we only analyzed baseline visit data. |
|  |  | (c) Consider use of a flow diagram | n/a | This was an historic observational cohort therefore would not be beneficial. |
| Descriptive data | 14* | (a) Give characteristics of study participants (eg demographic, clinical, social) and information on exposures and potential confounders | 8 | Baseline characteristics are noted in Table 1 in the manuscript. |
|  |  | (b) Indicate number of participants with missing data for each variable of interest | 4-5 | 765 participants were eligible for inclusion in our data analysis. 10 were excluded due to missing data from the primary outcome of self-reported AFib. |
|  |  | (c) *Cohort study*—Summarise follow-up time (eg, average and total amount) | 4 | All variables were collected at the baseline visit. |
| Outcome data | 15* | *Cohort study*—Report numbers of outcome events or summary measures over time | 7 | We evaluated 755 participants from the SCCOR cohort, of which 135 (17.9%) participants reported a history of AFib at the baseline visit (prevalent AFib). |
|  |  | *Case-control study—*Report numbers in each exposure category, or summary measures of exposure | n/a | This was a cohort study. |
|  |  | *Cross-sectional study—*Report numbers of outcome events or summary measures | n/a | This was a cohort study. |
| Main results | 16 | (*a*) Give unadjusted estimates and, if applicable, confounder-adjusted estimates and their precision (eg, 95% confidence interval). Make clear which confounders were adjusted for and why they were included | 6-7 | We used a 95% confidence interval for our analysis. The multivariable analysis was adjusted for the following: sex, age, number of pack years, BMI, self-reported heart failure, and anti-hypertensive medication use as these likely increase risk of AFib independently. |
|  |  | (*b*) Report category boundaries when continuous variables were categorized | 5, 9 | FEV_1_/FVC was grouped as <70% or > 70%. DLco% predicted was grouped as <60% or >60%. RV/TLC ratio was grouped as <40% or >40%. mMRC is scored on a scale between 0 and 4, with 4 indicating severe breathlessness. |
|  |  | (*c*) If relevant, consider translating estimates of relative risk into absolute risk for a meaningful time period | n/a | We did not determine relative or absolute risk in this study. |

Continued on next page

| Other analyses | 17 | Report other analyses done—eg analyses of subgroups and interactions, and sensitivity analyses | n/a | We did not do subgroup or sensitivity analyses. |
| --- | --- | --- | --- | --- |
| Discussion | | | | |
| Key results | 18 | Summarise key results with reference to study objectives | 9-10 | In the univariate analysis, DLco, heart volume, percentage of emphysema, and mMRC all showed statistically significant associations with prevalent AFib (Table 2). These associations remained significant when adjusting for sex, age, number of pack years, BMI, heart failure, and anti-hypertensive medication use. FEV₁ and the presence of airflow obstruction were not significantly associated with prevalent AFib. There was a statistically significant difference in mean CHA_2_DS_2_-VASc between those with and without prevalent AFib. For CHARGE-AF, there was no difference between the two groups (Table 3).  When conducting the discrimination analysis for prevalent AFib, the C-statistic was greater for CHA_2_DS_2_-VASc than CHARGE-AF, indicating it as a better risk predictor in our study population. This improved with the sequential addition of our exposure variables. Model 4 had the greatest improvement of discrimination, most notably for CHARGE-AF; this model incorporated DLco, heart volume, degree of emphysema, and mMRC score (Table 4 and Figure 1). |
| Limitations | 19 | Discuss limitations of the study, taking into account sources of potential bias or imprecision. Discuss both direction and magnitude of any potential bias | 13-14 | Our analysis has several limitations to note. AFib was assessed via self-report rather than through electronic health record (EHR) review or EKG documentation. Since AFib is often asymptomatic and paroxysmal, our number of participants with AFib was likely an under-estimate of the true prevalence and can be a source of measurement bias. There are also likely unmeasured confounding variables that we can’t account for. We did not include incident AFib in our analysis due to concern for variation in results secondary to two mechanisms, (1) a high dropout rate likely reducing the reported incident AFib cases and (2) those who developed incident AFib at a later timepoint were healthier with a higher FEV_1_, less pack year history, and fewer obstructive PFTs. Additionally, while we did not validate our findings in a separate cohort, this likely did not affect study results as the CHA_2_DS_2_-VASc and CHARGE-AF have already been validated in prior cohorts. Finally, our CT variables were created by an AI research algorithm and therefore may not be generalizable to a clinical population. Though we were able to evaluate chest CT scans, other radiographic studies like echocardiography or cardiac MRI were not available variables in the SCCOR cohort. Other variables, such as exacerbation history and active medication therapies could also be useful for analysis in future studies. |
| Interpretation | 20 | Give a cautious overall interpretation of results considering objectives, limitations, multiplicity of analyses, results from similar studies, and other relevant evidence | 11-14 | See discussion section for further details. We identified cardiopulmonary factors associated with an increased risk of AFib in a tobacco-exposed cohort. The incorporation of lung function, CT parameters, and symptom scores in validated AFib prediction models may improve AFib risk discrimination in our chronic lung disease populations. |
| Generalisability | 21 | Discuss the generalisability (external validity) of the study results | 13-14 | Our CT variables were created by an AI research algorithm and therefore may limit our generalizability secondary to logistical restraints. Additionally, our study only included former or current smokers. Otherwise, our study has excellent external validity as it reviewed real-world patients from a large academic center over a 10 year time period. |
| Other information | |  | | |
| Funding | 22 | Give the source of funding and the role of the funders for the present study and, if applicable, for the original study on which the present article is based | 15 | This study was supported by the National Institute of Health (NIH) National Heart, Lung and Blood Institute (NHLBI) grants 1R01HL128289 (J.B.) and P50HL084948 (F.C.S.). |

*Give information separately for cases and controls in case-control studies and, if applicable, for exposed and unexposed groups in cohort and cross-sectional studies.

**Note:** An Explanation and Elaboration article discusses each checklist item and gives methodological background and published examples of transparent reporting. The STROBE checklist is best used in conjunction with this article (freely available on the Web sites of PLoS Medicine at http://www.plosmedicine.org/, Annals of Internal Medicine at http://www.annals.org/, and Epidemiology at http://www.epidem.com/). Information on the STROBE Initiative is available at www.strobe-statement.org.
